# Qualitative and quantitative analyses of chemical components of Citri Sarcodactylis Fructus from different origins based on UPLC–Q–Exactive Orbitrap–MS and GC–MS

**DOI:** 10.1002/fsn3.2822

**Published:** 2022-03-14

**Authors:** Jinrong Ning, Kanghui Wang, Wanling Yang, Mengshi Liu, Jingyuan Tian, Minyan Wei, Guodong Zheng

**Affiliations:** ^1^ Key Laboratory of Molecular Target & Clinical Pharmacology and the State Key Laboratory of Respiratory Disease, School of Pharmaceutical Sciences & The Fifth Affiliated Hospital Guangzhou Medical University Guangzhou China

**Keywords:** Citri Sarcodactylis Fructus, GC–MS, qualitative and quantitative analyses, UPLC–Q–Exactive Orbitrap–MS

## Abstract

Ultra‐high‐performance liquid chromatography–Q–Exactive Orbitrap–mass spectrometry (MS) and gas chromatography (GC)–MS were performed for the qualitative and quantitative analyses of Citri Sarcodactylis Fructus (CSF) from different origins. The contents of eight major CSF components, namely 5,7‐dimethoxycoumarin, scopoletin, hesperidin, tangeretin, nobiletin, limonin, nomilin, and stachydrine, were quantitatively analyzed. Clustering analysis and principal component analysis (PCA) were, respectively, performed to classify and compare the 10 CSF batches. One hundred and two volatile components were identified accordingly by comparing retention times, reference standards, parent peaks, fragment peaks, and findings from relevant literature. Moreover, high content of 5,7‐dimethoxycoumarin and stachydrine was detected in all the CSFs, especially in CSF‐Zhe. Therefore, the high content component coumarin “5,7‐dimethoxycoumarin” was suggested to be quality analysis component rather than hesperidin. Additionally, characteristic compounds were found to distinguish different CSFs. This work was a comprehensive study about the components of various CSF. It distinguished the basic differences in the compositions of CSF from different origins. Eventually, it provided experimental and systematic bases for the quality control analysis of CSF, which has potential application in the further research.

## Graphical abstract

Ultra‐high‐performance liquid chromatography–Q–Exactive Orbitrap–mass spectrometry (MS) and gas chromatography (GC)–MS were performed for the qualitative and quantitative analyses of CSF from different origins. The contents of eight major CSF components, namely 5,7‐dimethoxycoumarin, scopoletin, hesperidin, tangeretin, nobiletin, limonin, nomilin, and stachydrine, were quantitatively analyzed. Principal component analysis (PCA) was performed to classify the 10 CSF batches.

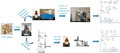

## 1 INTRODUCTION

Citri Sarcodactylis Fructus (CSF), the dry fruit of *Citrus medica* L. var. *sarcodactylis* Swingle with the Chinese name “Foshou”, belongs to Rutaceae family and is one of the most popular homology of medicine and food with tremendous medicinal value. In addition, CSF is an ornamental plant and its essential oil is used as a raw material for high‐grade spices, which has great economic prospects. CSF is divided into the following four groups according to the origin: CSF‐Guang (from Guangdong or Guangxi Province), CSF‐Zhe (from Zhejiang Province), CSF‐Chuan (from Sichuan Province), and CSF‐Yun (from Yunnan Province). CSF‐Guang and CSF‐Zhe have superior quality and are applied for the treatment of cough with phlegm, stomach distension, vomiting, and other diseases (Pharmacopoeia CON, 2020; Wang et al., 2012), which could be considered good development prospects. CSF shows essential pharmacological activities, such as gastroprotective (Zhu et al., 2020), anticancer (Fiorillo et al., 2018; Memariani et al., 2020), anti‐inflammatory (Carresi et al., 2020), anti‐oxidation (Hashemi & Jafarpour, 2020), antibacterial (Alexa et al., 2020), and neuroprotective activities (Rombolà et al., 2017, 2020).

Based on *Pharmacopoeia of the People's Republic of China* (ChP), hesperidin is the only CSF component with a content >0.030% and is therefore used in quality control. Considering that the hesperidin content of CSF is slightly low, hesperidin as a single indicator is not sufficient to distinguish CSFs from different origins. Therefore, scientific index components should be urgently explored via quantitative analysis.

Some CSF components have medicinal and commercial values (Chu et al., 2012; Yanmei et al., 2015; Zheng et al., 2021). In previous study, we have carried out a relatively comprehensive qualitative analysis of the nonvolatile components of CSF (Fu et al., 2020; Wang et al., 2021), including flavonoids, limonoids, organic acids, coumarins, and other compounds. However, the determination analysis of major pharmacological compositions and volatile oil that possesses remarkable potential value of CSFs from different regions is lacking. Few indicators of CSF quality are confirmed. Now this study will perform further quantitative analysis and clustering analysis of the CSF major components in order to make a systematic and comprehensive study on the CSF components.

Ultra‐high‐performance liquid chromatography (UPLC)–Q–Exactive Orbitrap–mass spectrometry (MS) (Arachchige et al., 2021; Mohd et al., 2021; Zheng et al., 2020) is often used for nonvolatile natural extracted product and gas chromatography (GC)–MS (Al‐Hwaiti et al., 2021; Ichihara et al., 2021; Tsurunaga et al., 2021) is used for volatile component analysis. They are considered reliable devices in CSF analysis owing to their rapidity and sensitivity in sample measurement.

In this work, a simple, effective, and precise method was established to comprehensively determine the compositions and distinction among CSFs from different origins.

## 2 MATERIALS AND METHODS

### 2.1 Materials and reagents

Ten CSF batches were harvested each from Guangdong or Guangxi Province (CSF‐Guang), Zhejiang Province (CSF‐Zhe), Sichuan Province or Chongqing municipality (CSF‐Chuan), and Yunnan Province (CSF‐Yun), China in 2019. The CSF samples were stored for 1 year and then used in the experiment. The detailed information of the CSF samples is shown in Table 1. All the samples were authenticated by Prof. Guodong Zheng (Guangzhou Medical University) and deposited in the Laboratory of Pharmacognosy, Guangzhou Medical University, Guangdong Province, China.

**TABLE 1:** Information of CSF samples from different origins

No.	Origins	Sample source	Collecting time	Extraction rate (%)
S1	CSF‐Guang	Le Town, Gaozong District, Zhaoqing City, Guangdong Province	25 October 2019	0.43
S2	CSF‐Guang	Yongfu County, Guilin City, Guangxi Province	28 October 2019	0.03
S3	CSF‐Guang	Yongfu County, Guilin City, Guangxi Province	29 October 2019	0.10
S4	CSF‐Zhe	Jinhua City, Zhejiang Province	28 October 2019	1.53
S5	CSF‐Zhe	Luodian Town, Wucheng District, Jinhua City, Zhejiang Province	7 November 2019	1.54
S6	CSF‐Zhe	Chisong Town, Jinhua City, Zhejiang Province	15 November 2019	1.66
S7	CSF‐Chuan	Huidong County, Liangshan Prefecture, Sichuan Province	21 October 2019	0.16
S8	CSF‐Chuan	Peng'an County, Nanchong City, Sichuan Province	17 November 2019	0.10
S9	CSF‐Yun	Qujing City, Yunnan Province	23 October 2019	0.04
S10	CSF‐Yun	Hualing County, Yuxi City, Yunnan Province	28 October 2019	0.39

The reference standards of stachydrine, scopoletin, hesperidin, 5,7‐dimethoxycoumarin, limonin, nomilin, nobiletin, and tangeretin were obtained from Weikeqi (Sichuan, China). The compounds had purities higher than 98%. High‐performance liquid chromatography (HPLC)‐grade formic acid was purchased from Thermo Fisher Scientific (USA). HPLC‐grade acetonitrile and *n*‐hexane were obtained from Merck (Germany). Deionized water was prepared by a Milli‐Q system (Millipore, USA).

### 2.2 Sample and standard preparation

For UPLC–Q–Exactive Orbitrap–MS analysis, the samples were crushed and passed through a 40‐mesh sieve to obtain a small powder. Each powdered sample (0.1 g) was precisely weighed, soaked with 50 ml of methanol, and ultrasonicated for 30 min (320 W, 40 kHz). After the samples were cooled to room temperature, methanol was added to the CSF extracts until the weight was the same as its initial weight. The filtrate was prepared and then condensed to 5 ml by a XHRE‐2000C rotary evaporator (Shanghai Xiaohan Co., LTD., China). After filtering through a 0.22‐μm microporous membrane, the obtained sample solution was injected into the UPLC–Q–Exactive Orbitrap–MS equipment. The standards of stachydrine, scopoletin, hesperidin, 5,7‐dimethoxycoumarin, limonin, nomilin, nobiletin, and tangeretin were accurately weighed and dissolved in 50 ml of methanol. Then, methanol was used to dilute the mixture solution to obtain the concentrations of the tested compounds, which were filtered by a 0.22‐μm microporous membrane for UPLC–Q–Exactive Orbitrap–MS. Then, method validation was performed to determine the repeatability, stability, precision, recovery, and linear correlation of the method.

For GC–MS analysis, each sample was smashed into coarse powder, and approximately 60 g of the crushed powder was weighed in a round‐bottom flask. The mixture was added with 600 ml of deionized water, fully soaked for 12 hr, and heated for 5 hr. Then, volatile oil was flowed back into the extractor with water vapor and then collected at room temperature. The obtained volatile oil was stored at 4°C in the dark to prevent degradation. The weight of the extracted volatile oil and tested compounds were used to calculate the extraction rate in volatile oil. The extracted volatile oil (50 μL) was diluted 20 times with *n*‐hexane, filtered through a 0.22‐μm microporous membrane, and then injected into the GC–MS system.

### 2.3 UPLC–Q‐Exactive Orbitrap–MS determination analysis

UPLC analysis was performed on a Dionex Ultimate 3000 series UPLC system equipped with an online degasser, a column temperature compartment, a quaternary pump, and an auto‐sampler manager (Thermo Fisher Scientific, USA). Chromatographic separation was performed on a ZORBAX Eclipse Plus C_18_ column (2.1 mm × 50 mm, 1.8 μm; Agilent, USA) at 35 ℃. The mobile phase was composed of 0.05% (v/v) water–formic acid (A) and acetonitrile (B) with the following gradient elution conditions: 0–5 min, 10% B; 5–10 min, 10%–20% B; 10–20 min, 20%–50% B; 20–30 min, 50%–85% B; 30–40 min, 85%–100% B (Wang et al., 2021). The flow rate was sustained at 1.0 ml/min with an injection volume of 10 μl.

MS identification was integrated on a Q‐Exactive Orbitrap–tandem MS instrument (Thermo Fisher Scientific, USA). Positive ion mode with 3500 V ion spray voltage was used. Nitrogen was applied as sheath gas (30 units), auxiliary gas (10 units), and sweep gas (5 units). Ion source temperature was sustained at 320 ℃, and desolvation temperature was sustained at 300 ℃. Full mass scan was set in the range of m/z 100–1000 Da. All the chemical compounds were discriminated by contrasting the mass spectra with the standard reference, reported literature, and Orbitrap Chinese Traditional Medicine Library (OCTML).

### 2.4 GC–MS analysis

GC analysis was performed on a TRACE DSQ GC instrument (Thermo Finnigan, USA). TG‐5SILMS GC capillary column (0.25 mm × 30 m, 0.25 μm; Thermo Fisher Scientific, USA) was applied, and the temperature program was set as follows: initially, the temperature was set at 60 ℃ for 3 min, then increased to 80 ℃ at 1 ℃·min^‒1^ for 3 min, further increased to 250 ℃ at 10 ℃·min^‒1^, and finally increased to 300 ℃ at a rate of 25 ℃·min^‒1^ for 5 min. High‐purity helium, the carrier gas, was operated at a flow rate of 1.0 ml·min^‒1^, and 1 μl of the sample solution was injected in a splitting ratio at 50:1.

MS identification was operated on a TRACE DSQ mass spectrometer (Thermo Finnigan, USA). Ionization energy was maintained at 70 eV, and temperature in the electron‐impact ion source was set to 270 ℃. The mass scan ranged from m/z 35 to 500 amu, and solvent delay time was set to 4 min. All the constituents in volatile oil were identified based on the comparison of the mass spectra and ion fragmentation with reported literature and MS library (NIST 2008).

### 2.5 Statistical analysis

All the data of CSF from different origins were statistically analyzed using SPSS and GraphPad Prism software. Clustering analysis and principal component analysis were applied to observe the similarities and differences of the CSFs from different origins.

## 3 RESULTS AND DISCUSSION

### 3.1 Chemical determination of CSF

**FIGURE 1:**
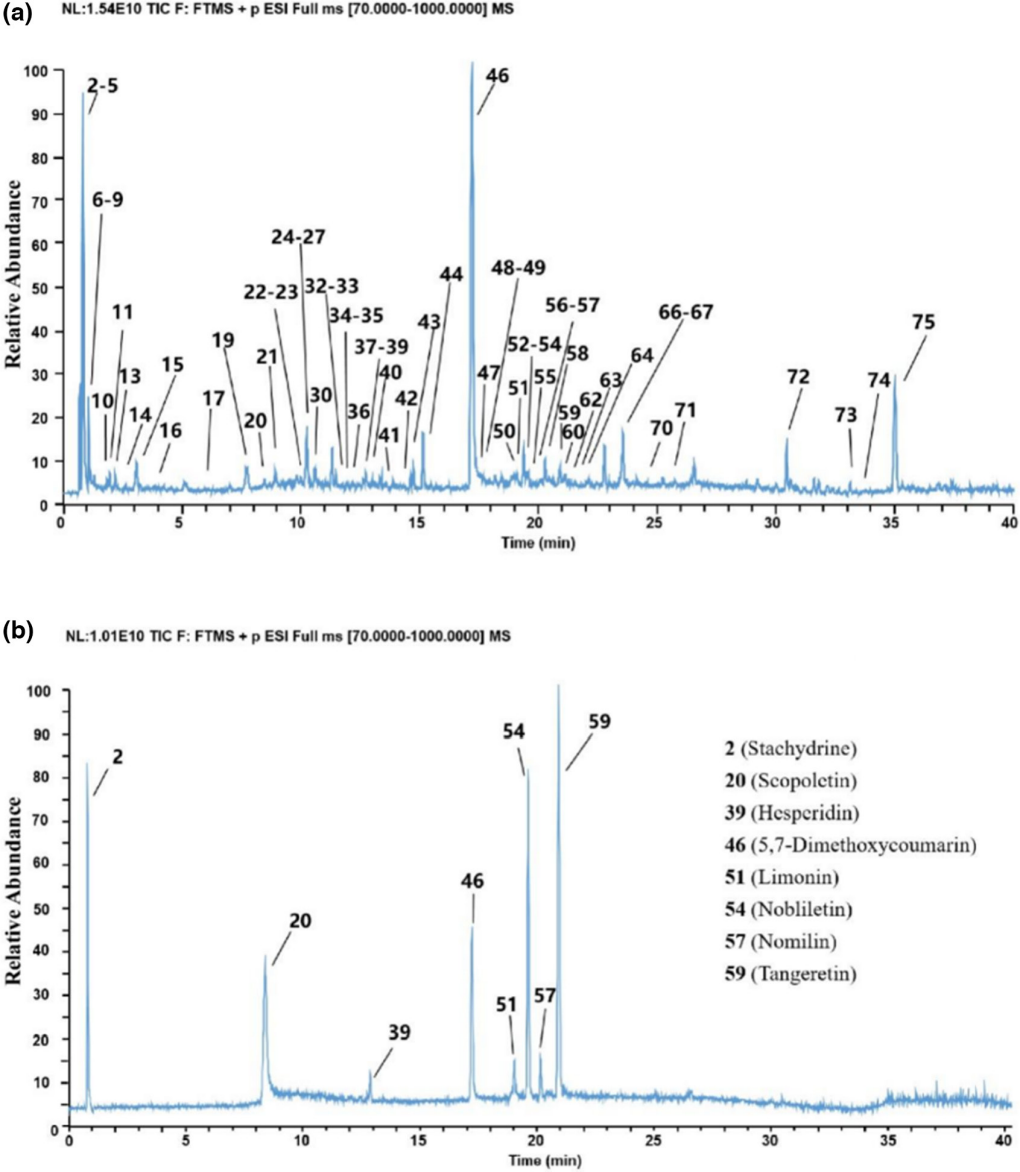
Representative total ion chromatograms of nonvolatile compounds in (A) CSF and (B) mixed standards

Based on the previous study, the methanol extracts of 10 batches of CSFs from various geographical origins were separated and analyzed by UPLC–Q–Exactive Orbitrap–MS. The total ion chromatograms (TICs) of the methanol extract and mixed reference substances in positive ion mode are shown in Figure 1. The components were separated in 40 min under the gradient elution program of this experiment. Eight main components of CSF, namely, 5,7‐dimethoxycoumarin, scopoletin, limonin, nomilin, stachydrine, hesperidin, tangeretin, and nobiletin, were detected and determined the concentration by UPLC–Q–Exactive Orbitrap–MS (Table 2).

**TABLE 2:** Information of the eight compounds

Structure	No.	Name	t_R_(min)	[M + H]^+^(m/z)	Compound formula
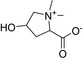	2	Stachydrine	0.83	144.1018	C_7_H_13_O_2_N
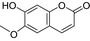	20	Scopoletin	8.35	193.0498	C_10_H_8_O_4_
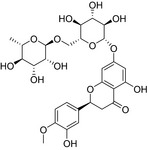	39	Hesperidin	12.85	611.1964	C_28_H_34_O_15_
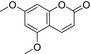	46	5,7‐Dimethoxycoumarin	17.2	207.0654	C_11_H_10_O_4_
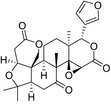	51	Limonin	18.99	471.2013	C_26_H_30_O_8_
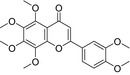	54	Nobiletin	19.61	403.1388	C_21_H_22_O_8_
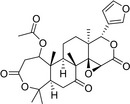	57	Nomilin	20.14	515.2272	C_28_H_34_O_9_
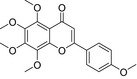	59	Tangeretin	20.91	373.1286	C_20_H_20_O_7_

### 3.2 Method validation

Good linear correlations were obtained for the eight compounds using this method with *R* ^2^>0.995 (Table 3). The limit of detection and limit of quantification were the mass concentrations of the compound when the signal‐to‐noise ratios were 3 and 10, respectively. Moreover, the relative standard deviations of the repeatability (0.47%–3.00%), precision (0.74%–2.0%), stability (0.49%–2.88%), and recovery (0.31%–2.86%) of the method were all below 3.00%, and the recovery was within the range of 92.58%–108.98%. These results demonstrated that the UPLC–Q–Exactive Orbitrap–MS method was effective and accurate for the quantitative analysis of CSF composition.

**TABLE 3:** Methodological data of eight standards

Compound	Regression equation	R^2^	Repeatability RSD(%),*n* = 6	Precision RSD(%),*n* = 6	Stability RSD(%),*n* = 6	Recovery(%), *n* = 6	Recovery RSD(%), *n* = 6
5,7‐Dimethoxycoumarin	y = 118,269,738 x + 17,933,334,241	0.999	0.47	0.74	0.49	99.76	0.31
Nobiletin	y = 228,938,807 x + 48,230,514	1.000	2.89	1.95	1.45	99.93	0.57
Limonin	y = 30,145,653 x + 129,121,002	1.000	2.24	1.62	2.88	92.58	0.25
Nomilin	y = 7,284,725 x + 158,829,053	0.999	3.00	2.00	2.35	102.10	0.75
Tangeretin	y = 227,402,654 x + 57,630,304	0.999	3.00	2.00	2.75	108.98	0.90
Scopoletin	y = 168,250,777 x + 4,207,016	0.999	0.76	0.88	1.06	99.33	1.08
Stachydrine	y = 47,933,086 x + 6,222,407,943	0.995	1.47	1.23	2.74	96.16	1.57
Hesperidin	y = 3,587,338 x + 7,521,407	0.999	2.31	1.66	1.73	103.53	2.86

### 3.3 Concentrations of CSF components

The contents of the eight principal components in the 10 batches of CSF (Table 4) were obtained by the proposed standard method to obtain a better description of CSFs from different origins. The result indicated that the 10 CSF batches contained high levels of 5,7‐dimethoxycoumarin (0.631–3.613 mg·g^−1^), stachydrine (1.261–1.555 mg·g^−1^), and nomilin (0.261–0.769 mg·g^−1^) and extremely low levels of hesperidin (0.044–0.232 mg·g^−1^), scopoletin (0.003–0.018 mg·g^−1^), limonin (0.025–0.084 mg·g^−1^), tangeretin (0.004–0.083 mg·g^−1^), and nobiletin (0.003–0.050 mg·g^−1^). Compared with major CSF cultivars, CSF‐Zhe contained the most abundant content of 5,7‐dimethoxycoumarin at 2.730–3.3613 mg·g^−1^. Additionally, hesperidin concentration in CSF‐Zhe was higher than those in the three other CSFs. The tangeretin contents of CSF‐Zhe and CSF‐Guang were relatively higher than those CSF‐Chuan and CSF‐Yun. However, nomilin was relatively higher in CSF‐Yun than in other CSFs.

**TABLE 4:** Contents of eight CSF components in different CSF samples

No.	Content (mg·g^−1^)							
	5,7‐Dimethoxycoumarin	Scopoletin	Limonin	Nomilin	Stachydrine	Hesperidin	Tangeretin	Nobiletin
S1	0.808 ± 0.004	0.008 ± 0	0.084 ± 0.001	0.645 ± 0.016	1.555 ± 0.028	0.224 ± 0.003	0.083 ± 0.001	0.050 ± 0.001
S2	1.949 ± 0.008	0.003 ± 0	0.065 ± 0	0.390 ± 0.002	1.277 ± 0.026	0.095 ± 0.001	0.042 ± 0	0.021 ± 0
S3	1.524 ± 0.003	0.005 ± 0	0.068 ± 0	0.441 ± 0.007	1.347 ± 0.020	0.094 ± 0.002	0.012 ± 0	0.005 ± 0
S4	3.290 ± 0.006	0.005 ± 0	0.050 ± 0	0.164 ± 0.001	1.331 ± 0.019	0.132 ± 0.002	0.040 ± 0	0.015 ± 0
S5	2.730 ± 0.013	0.018 ± 0	0.074 ± 0	0.325 ± 0.003	1.435 ± 0.009	0.141 ± 0.002	0.033 ± 0	0.020 ± 0
S6	3.613 ± 0.018	0.010 ± 0	0.077 ± 0.001	0.498 ± 0.005	1.311 ± 0.020	0.232 ± 0.002	0.023 ± 0	0.011 ± 0
S7	1.192 ± 0.004	0.014 ± 0	0.062 ± 0.001	0.504 ± 0.004	1.277 ± 0.012	0.039 ± 0.001	0.008 ± 0	0.006 ± 0
S8	1.566 ± 0.006	0.014 ± 0	0.025 ± 0	0.261 ± 0.002	1.459 ± 0.005	0.188 ± 0.004	0.009 ± 0	0.008 ± 0
S9	0.631 ± 0.005	0.005 ± 0	0.073 ± 0	0.743 ± 0.014	1.391 ± 0.018	0.133 ± 0.004	0.005 ± 0	0.003 ± 0
S10	1.098 ± 0.004	0.008 ± 0	0.070 ± 0.001	0.769 ± 0.018	1.261 ± 0.026	0.044 ± 0.001	0.004 ± 0	0.003 ± 0

Hesperidin is a flavonoid substance that is widely found in citrus and contains substantial pharmacological activities, such as anti‐neoplastic, anti‐oxidation, and anti‐inflammatory activities; its content has been detected as a biomarker for quality control in CSF as described in ChP (2020). The present study indicated that hesperidin had extraordinarily low content and low response value compared with the other main components; therefore, it is not efficient and suitable as an index for the estimation of CSF quality. In comparison, 5,7‐dimethoxycoumarin has remarkable pharmacological activity (Alesiani et al., 2009; Yang & Wang, 2018) and is therefore more suitable for CSF quality control.

**FIGURE 2:**
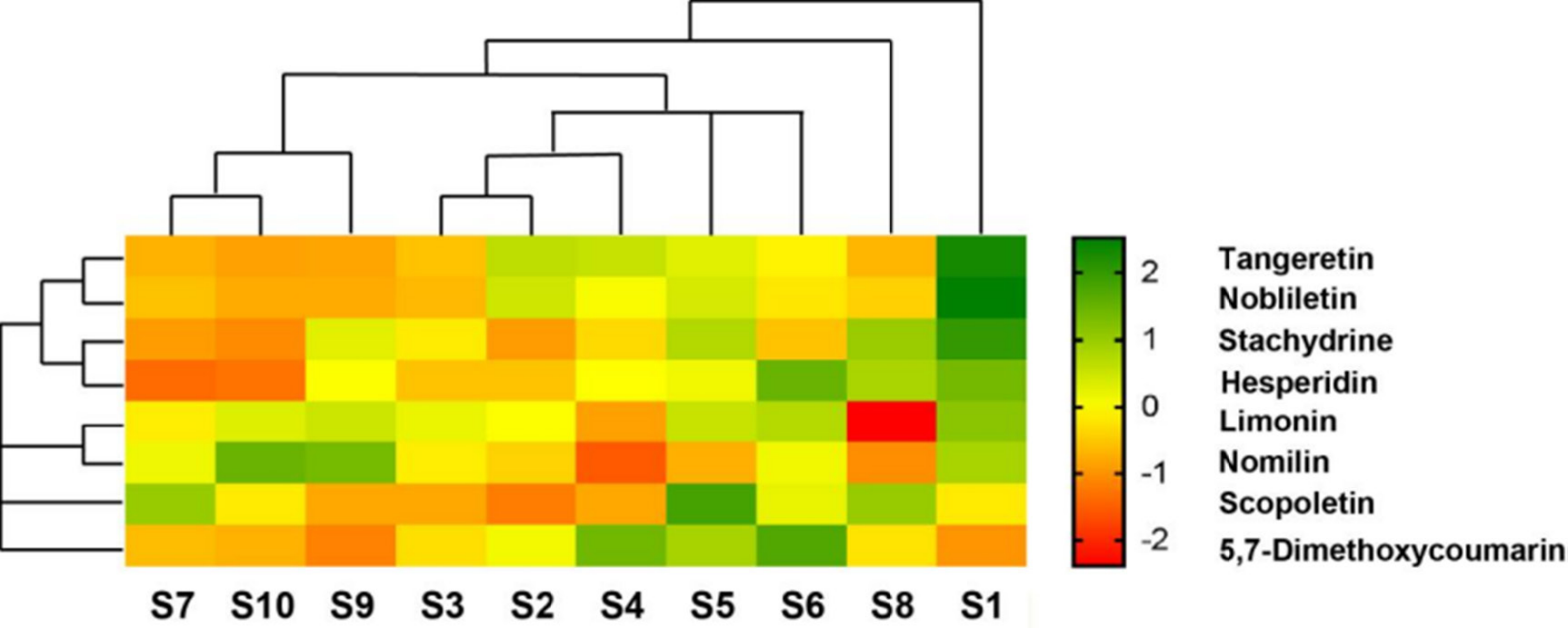
Heat map and dendrogram of CSF components from different CSF samples

The contents of eight main CSF components were subjected to clustering analysis to determine the similarity among CSFs from different origins. The heat map and dendrogram of eight CSF components are shown in Figure 2. The results manifested that CSF‐Zhe and CSF‐Guang (S2 and S3) were classified together, and CSF‐Yun and CSF‐Chuan (S7) were classified together.

### 3.4 GC–MS analysis of CSF

**FIGURE 3:**
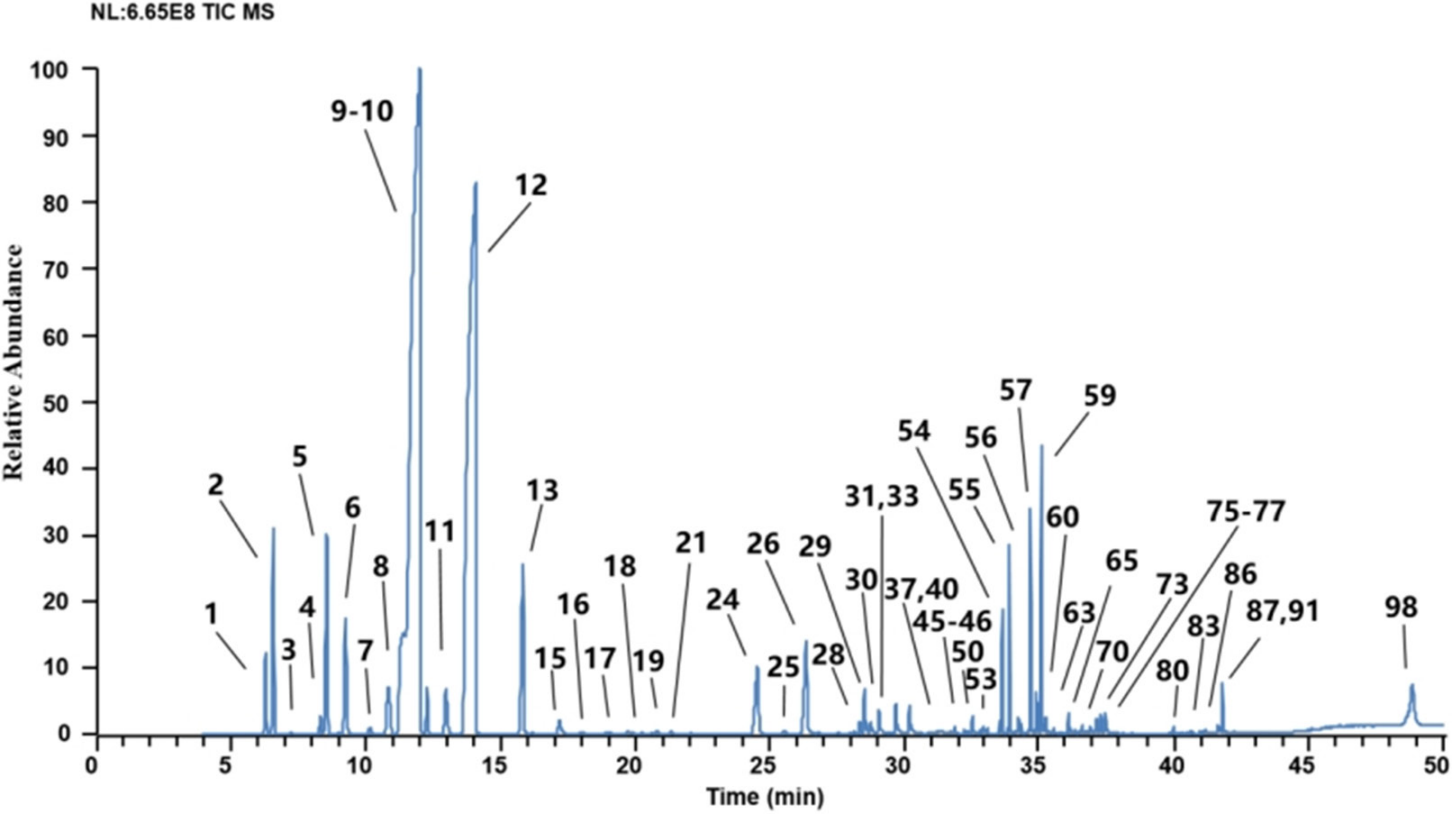
Representative total ion chromatograms of volatile components in CSF

GC–MS analysis detected 102 kinds of volatile oil from the CSF samples. The volatile oils included 5 alkanes, 38 alkenes, 24 alcohols, 5 ketones, 4 aldehydes, 2 phenols, 20 esters, and 4 other compounds (Table 5). The TIC of the methanol extraction is shown in Figure 3.

**TABLE 5:** Summary of volatile compounds in CSF identified by GC–MS

No.	t_R_ (time)	Compound	S1	S2	S3	S4	S5	S6	S7	S8	S9	S10
Alkane												
23	22.69	endo−2‐Chlorobornane	—	—	—	0.01	—	—	—	—	—	—
37	31.30	2‐Isopropenyl−5‐methylhex−4‐enal	0.07	—	—	—	0.03	—	—	—	—	—
41	31.47	3,6,7‐Trioxatricyclo[3.2.2.02,4]nonane, 1‐methyl−5‐(1‐methylethyl)‐	—	—	—	—	—	0.02	—	—	—	—
86	41.25	Pentacyclo[9.1.0.0(2,4).0(5,7).0(8,10)]dodecane,3,3,6,6,9,9,12,12‐octamethyl‐,anti,anti,anti‐	0.07	0.08	0.07	—	—	—	—	—	—	0.19
98	48.92	Tetratetracontane	1.43	—	—	—	—	—	—	—	—	—
Alkene												
1	6.37	Thujene	0.90	0.30	0.33	0.76	0.99	0.89	0.13	0.24	0.33	0.29
2	6.64	ɑ‐Pinene	2.43	0.99	1.20	2.37	2.78	2.59	0.55	1.11	1.05	1.11
3	7.31	Camphene	0.03	—	—	0.03	0.03	0.03	—	—	—	—
4	8.40	Sabinene	0.24	0.06	0.09	0.18	0.21	0.20	0.03	0.28	0.07	0.09
5	8.62	β‐Pinene	2.94	0.97	1.63	2.55	2.91	2.67	0.71	1.43	0.67	1.44
6	9.33	β‐Myrcene	1.87	0.72	0.98	1.75	2.13	1.93	0.36	1.06	0.51	1.34
7	10.23	α‐Phellandrene	0.15	0.06	0.09	0.10	0.12	0.11	0.09	0.12	—	0.09
8	10.92	α‐Terpinene	1.24	0.46	0.71	0.83	0.88	0.78	0.54	0.43	0.66	0.59
10	12.05	D‐Limonene	42.31	41.25	37.31	52.08	54.33	53.15	38.59	43.60	50.25	50.15
11	12.35	(E)‐β‐Ocimene	1.37	0.65	1.00	2.50	3.43	2.84	0.59	1.23	—	0.98
12	14.11	γ‐Terpinene	31.73	21.31	26.45	26.62	27.02	26.26	23.03	22.53	10.63	22.74
13	15.89	Terpinolene	2.43	0.46	0.71	—	—	—	—	—	—	—
14	16.22	p‐Cymene	—	—	—	—	—	—	—	0.09	—	—
16	18.10	1,3,8‐p‐Menthatriene	0.05	0.10	0.18	0.01	0.02	0.02	—	0.28	—	0.12
18	19.80	Neoalloocimene	0.10	—	—	0.08	0.12	0.11	—	0.05	—	—
39	31.44	Cyclohexene, 2‐ethenyl−1,3,3‐trimethyl‐	—	—	0.13	—	—	—	—	—	—	0.12
40	31.46	Ascaridole epoxide	0.08	—	—	—	—	—	—	—	—	—
46	31.93	γ‐Elemene	0.41	1.16	0.93	0.33	0.25	0.21	0.24	0.12	0.57	0.53
47	31.99	Cyclohexene,4‐ethenyl−4‐methyl−3‐(1‐methylethenyl)−1‐(1‐methylethyl)‐, (3R‐trans)‐	—	—	—	—	—	—	0.16	0.08	0.71	0.66
48	32.25	α‐Cubebene	—	0.05	0.11	0.04	0.02	0.02	0.07	—	—	—
51	32.84	Copaene	—	—	—	—	0.02	0.02	0.10	—	—	—
53	33.16	β‐Elemene	0.06	0.13	0.11	0.06	0.04	0.04	0.16	0.11	0.18	0.19
54	33.70	Caryophyllene	0.80	2.77	2.54	0.90	0.85	0.80	2.26	1.17	1.91	1.05
55	33.96	α‐Bergamotene	1.07	2.56	1.95	0.68	0.72	0.68	2.84	1.72	2.37	1.73
56	34.30	α‐Caryophyllene	0.22	0.59	0.51	0.16	0.17	0.14	0.46	0.30	0.45	0.31
57	34.75	Germacrene D	1.29	3.03	2.83	1.07	0.85	0.81	3.79	1.01	1.25	0.96
58	35.00	Bisabolene	—	—	—	—	—	—	—	0.32	—	—
59	35.14	6‐methyl−2‐(4‐methylcyclohex−3‐enyl)hept−1,5‐diene	1.62	4.02	3.08	0.99	1.04	0.95	3.84	2.49	1.69	2.17
60	35.31	d‐Cadinene	0.10	0.35	0.30	0.09	0.09	0.08	0.73	0.16	0.52	0.10
63	35.61	(Z)‐α‐Bisabolene	0.03	0.09	0.07	0.01	—	—	—	0.05	—	0.05
64	35.87	γ‐Elemene	—	—	—	—	—	—	0.10	—	0.36	0.31
66	36.25	Guaiene	—	—	—	—	—	—	—	—	—	0.07
68	36.52	Aromadendrene oxide	—	—	0.06	—	—	—	—	—	—	—
69	36.64	a‐cis‐Himachalene	—	0.23	0.15	—	—	—	—	—	—	0.07
91	41.88	Neoisolongifolene, 8‐bromo‐	0.31	0.87	0.67	—	—	—	0.41	—	1.27	1.35
95	47.37	7‐[(Trimethylsilyl)oxy]androst−4‐ene−3,17‐dione bis(O‐methyloxime)	—	—	—	—	—	—	0.07	—	—	—
99	49.19	Dihydrofuro[2,3‐e]phenalene,6‐methoxy−8‐oxo−2,3,3,10‐tetramethyl−4,5,7‐trihydroxy	—	—	—	—	—	—	—	—	0.23	—
101	49.47	Pregn−4‐ene−3,20‐dione, 11,17‐bis[(trimethylsilyl)oxy]‐, bis(O‐methyloxime), (11beta)‐	—	—	—	—	—	—	—	—	0.22	—
Alcohol												
15	17.24	Linalool	0.31	0.27	0.35	0.12	0.13	0.14	0.23	0.41	—	0.44
17	19.14	2‐Cyclohexen−1‐ol, 1‐methyl−4‐(1‐methylethyl)‐,cis‐	0.05	—	0.11	0.02	0.03	0.03	0.10	—	—	—
19	20.89	Thujyl alcohol	0.08	—	—	—	—	—	—	—	—	—
24	24.54	L−4‐Terpineol	1.73	1.74	2.06	0.81	0.85	0.90	2.79	1.72	1.02	1.17
25	25.60	p‐Cymenol	0.08	—	0.05	0.01	0.03	0.03	—	0.12	—	—
26	26.42	α‐Terpineol	2.20	2.31	2.62	1.27	1.17	1.33	3.00	3.15	1.93	2.09
27	28.22	Carveol	—	—	—	—	0.01	—	—	0.06	—	—
28	28.41	trans−3‐Caren−2‐ol	0.13	0.09	0.16	0.03	0.04	0.06	—	0.07	—	0.09
31	28.83	Citronellol	0.18	0.13	0.15	—	—	—	—	0.14	—	0.23
33	29.12	Nerol	0.91	0.25	0.56	0.01	0.04	0.09	0.23	0.76	0.01	1.88
38	31.31	Cyclopropanemethanol,2‐methyl−2‐(4‐methyl−3‐pentenyl)‐	—	—	—	—	—	0.02	—	—	—	—
61	35.39	Bergamotenol	—	—	—	—	—	—	—	—	—	0.05
62	35.49	Cedran‐diol, 8S,14‐	—	—	—	—	—	—	—	—	0.17	—
65	36.16	(‐)‐Spathulenol	0.16	0.22	0.38	0.09	0.15	0.18	0.90	0.80	1.17	0.12
67	36.37	Himbaccol	—	0.11	0.07	0.01	0.09	0.09	0.13	0.07	—	—
71	36.76	1,3a‐Ethano(1H)inden−4‐ol,octahydro−2,2,4,7a‐tetramethyl‐	—	0.14	—	0.03	—	—	—	—	—	—
73	36.99	t‐Cadinol	0.09	0.12	0.20	0.02	0.03	0.04	0.24	0.13	0.22	0.05
74	37.18	Ledol	—	0.45	0.45	—	—	—	0.52	0.29	—	0.20
76	37.33	(‐)‐Isolongifolol	0.12	0.43	0.41	0.06	0.07	0.07	0.36	0.25	0.27	0.21
77	37.53	α‐Bisabolol	0.19	0.60	0.54	0.09	0.13	0.13	0.81	0.44	0.45	0.29
81	40.63	1‐Heptatriacotanol	—	0.10	—	—	—	—	—	—	—	—
82	40.63	FW−306	—	—	0.09	—	—	—	—	—	—	0.15
83	40.68	Cycloeucalenol	0.04	—	—	—	—	—	—	—	—	—
89	41.86	Urs−12‐en−28‐ol	—	—	—	0.04	—	—	—	—	—	—
Ketone												
20	20.97	Camphor	—	—	—	0.03	0.03	0.03	—	—	—	—
21	21.42	1‐(1,4‐dimethyl−3‐cyclohexen−1‐yl)‐Ethanone	0.07	0.10	0.10	0.01	—	0.03	0.08	0.08	—	0.05
34	29.61	Piperitone	—	—	—	0.01	—	—	—	—	—	—
94	45.91	3,9β,14,15‐Diepoxypregn−16‐en−20‐one,3,11β,18‐triacetoxy‐	—	0.10	—	—	—	—	—	—	—	—
97	48.04	(1aR)−3‐(Acetyloxymethyl)−9β,9aα‐bis(acetyloxy)−1,1aα,1bβ,4,4aβ,7aα,7b,8,9,9a‐decahydro−7bα‐hydroxy−1,1,6,8α‐tetramethyl−5H‐cyclopropa[3,4]benz[1,2‐e]azulen−5‐one	—	—	—	—	—	—	0.16	—	—	—
Aldehyde												
29	28.58	2,6‐Octadienal, 3,7‐dimethyl‐, (Z)‐	0.64	0.25	1.44	0.11	0.11	0.19	—	0.46	—	1.34
30	28.82	Citronellal	0.05	—	—	—	—	—	—	—	—	—
32	28.88	2‐Isopropenyl−5‐methylhex−4‐enal	—	—	—	0.02	0.03	—	—	—	—	—
43	31.66	3,5‐Heptadienal, 2‐ethylidene−6‐methyl‐	—	—	—	0.02	—	—	—	—	—	—
Phenol												
42	31.49	Thymol	—	—	—	—	0.02	0.02	—	0.16	—	—
70	36.67	Cubenol	0.06	0.13	0.17	0.03	0.05	0.07	0.18	0.11	0.15	0.06
Ester												
22	21.55	Isopulegol acetate	—	—	—	—	0.01	—	—	—	—	—
44	31.75	3,7‐Nonadien−2‐ol,4,8‐dimethyl‐, 2‐acetate	—	—	0.05	—	—	—	—	—	—	—
45	31.78	Methylgeranate	0.03	—	—	—	—	—	—	—	—	—
49	32.37	Rhodinyl acetate	—	—	—	—	—	—	—	0.13	—	0.06
50	32.59	Neryl acetate	0.26	0.11	0.09	0.04	0.04	0.05	0.21	2.30	0.27	1.08
52	32.84	2,6,10‐Dodecatrien−1‐ol, 3,7,11‐trimethyl‐, acetate, (E,E)‐	—	—	—	—	—	—	—	—	0.43	—
72	36.76	Yohimbine	—	—	—	—	0.04	—	—	—	—	—
75	37.20	Bicyclo[2.2.1]heptane,2‐methyl−3‐methylene−2‐(4‐methyl−3‐pentenyl)‐, (1S‐endo)‐	0.13	—	—	0.08	—	—	—	—	0.42	—
78	37.85	Methyl tetradecanoate	—	0.13	—	—	—	—	—	—	—	—
79	37.89	Terpinyl propionate	—	—	0.12	—	—	—	—	—	—	0.24
80	40.06	Methyl palmitate	0.07	0.22	0.24	0.01	0.04	0.03	0.19	0.27	0.13	0.20
84	40.77	1‐O‐Linoleoyl−2‐O,3‐O‐bis(trimethylsilyl)glycerol	—	—	—	—	—	—	0.07	—	0.33	—
85	41.23	2‐Butenoic acid, 2‐methyl‐,2‐(acetyloxy)−1,1a,2,3,4,6,7,10,11,11a‐decahydro−7,10‐dihydroxy−1,1,3,6,9‐pentamethyl−4a,7a‐epoxy−5H‐cyclopenta[a]cyclopropa[f]cycloundecen−11‐yl ester	—	—	—	—	—	—	—	—	0.22	—
87	41.71	Methyl octadeca−9,12‐dienoate	0.10	0.50	0.47	—	0.07	—	0.19	0.40	—	0.24
88	41.76	Methyl linolenate	—	—	—	—	—	0.03	—	—	—	—
90	41.86	Gitoxigenin	—	—	—	—	—	0.02	—	—	—	—
92	42.28	Ethyl linoleate	—	0.09	0.08	—	—	—	—	0.05	—	0.06
93	45.77	1,6‐Octadien−3‐ol, 4,7‐dimethyl‐, isovalerate	—	—	—	—	—	—	—	—	—	0.06
96	47.58	Octadecanoic acid 3‐octadecyloxypropyl ester	—	—	—	—	—	—	0.08	—	—	—
102	49.61	3'H‐Cycloprop[1,2] androst−1‐ene−3',3'‐dicarboxylic acid, 17‐(acetyloxy)−1,2‐dihydro−17‐methyl−3‐oxo‐, 3',3'‐diethyl ester, (1β,2β,5α,17β)‐	—	—	—	—	—	—	—	—	0.12	—
Other compound												
9	11.37	p‐Cymene	4.78	6.24	3.42	6.27	6.02	5.14	2.60	7.28	4.31	2.67
35	29.79	24, 25‐Dihydroxy VD3	—	—	—	—	—	—	—	—	0.11	—
36	29.91	Geranyl vinyl ether	—	—	—	—	0.01	—	—	—	—	—
100	49.45	6‐AH‐cAMP	—	—	—	—	—	0.03	—	—	—	—

Consequently, the result revealed that CSF contained abundant γ‐terpinene (10.63%–31.73%) and D‐limonene contents (37.31%–54.33%), which possess excellent antifungal (Karpiński, 2020), anti‐inflammatory (Ramalho et al., 2015), and antioxidant activities (Guo et al., 2021). The extraction rates of volatile oils are shown in Table 1. In this case, CSF‐Zhe contained the most content of D‐limonene (52.08%–54.33%). Moreover, CSF‐Zhe (1.53%–1.66%) had the largest extraction rate of volatile content compared with the other tested samples (0.03%–0.43%). CSF‐Zhe and CSF‐Guang had higher γ‐terpinene content, and CSF‐Zhe possessed the highest D‐limonene content. Tetratetracontane (1.46%) was only detected in CSF‐Guang (S1), whereas terpinolene (0.71%–2.43%) was only found in CSF‐Zhe. Tetratetracontane, the exclusive component in S1, may be the characteristic compound to distinguish CSF‐Guang (Guangdong Province) from the other CSFs. Terpinolene may be used as a biomarker to discriminate CSF‐Zhe from the other CSFs.

**FIGURE 4:**
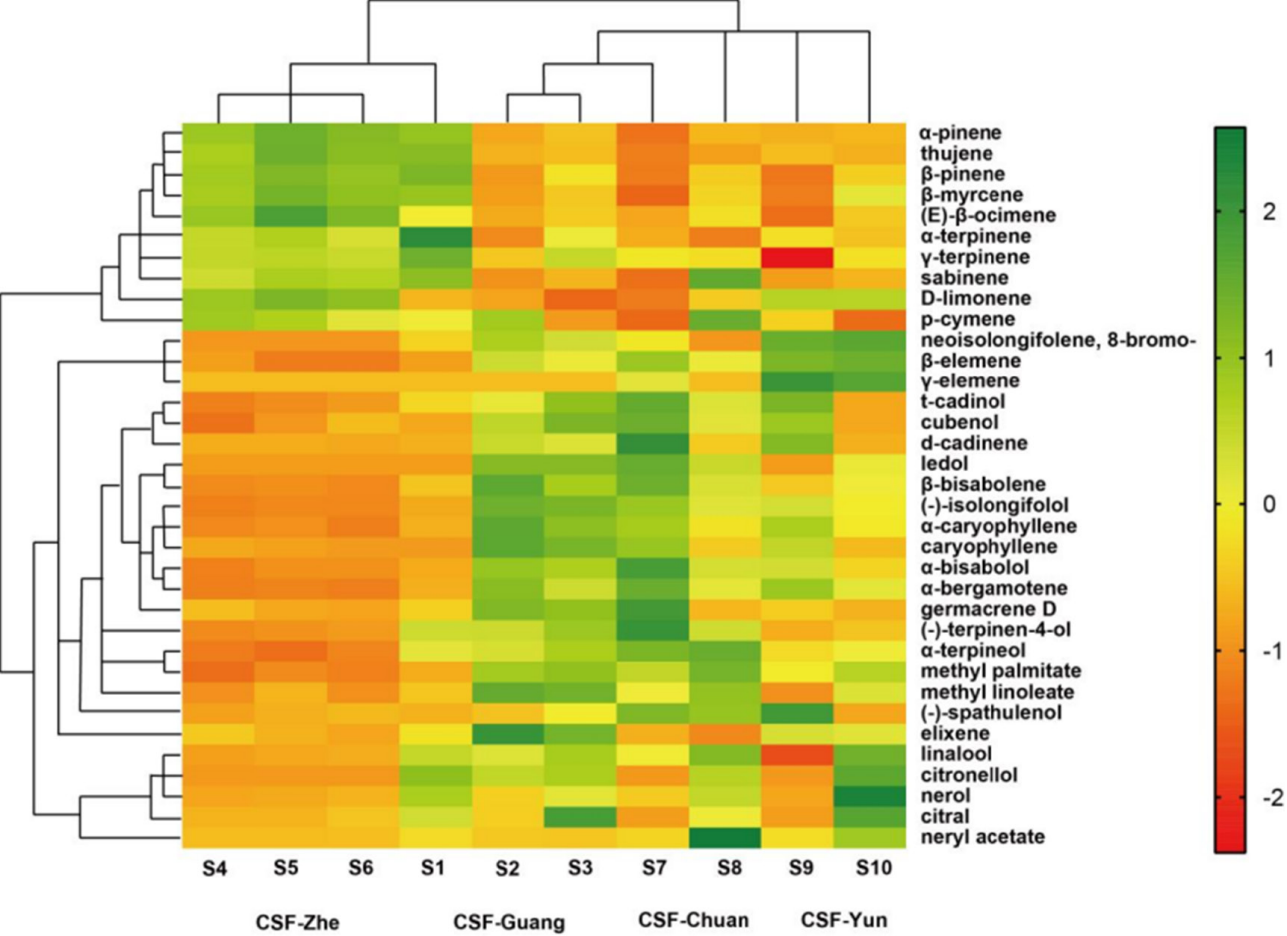
Heat map and dendrogram of 35 volatile oils in CSF from different origins

To classify the relativity among the tested CSFs by their chemical uniqueness, 35 main volatile components were standardized and performed clustering analysis. The heat map of the relative peak area based on 35 main volatile components is shown in Figure 4. In this experiment, CSF‐Zhe and CSF‐Guang (S1) were classified into one category, CSF‐Guang (S2 and S3), CSF‐Chuan, and CSF‐Yun were classified into another category. Based on the 35 main volatiles, CSF‐Zhe and CSF‐Guang (S1) shown chemical similarities, which contain higher content of ɑ‐pinene, thujene, β‐pinene, β‐myrcene, (E)‐β‐ocimene, α‐terpinene, γ‐terpinene, and sabinene.

**FIGURE 5:**
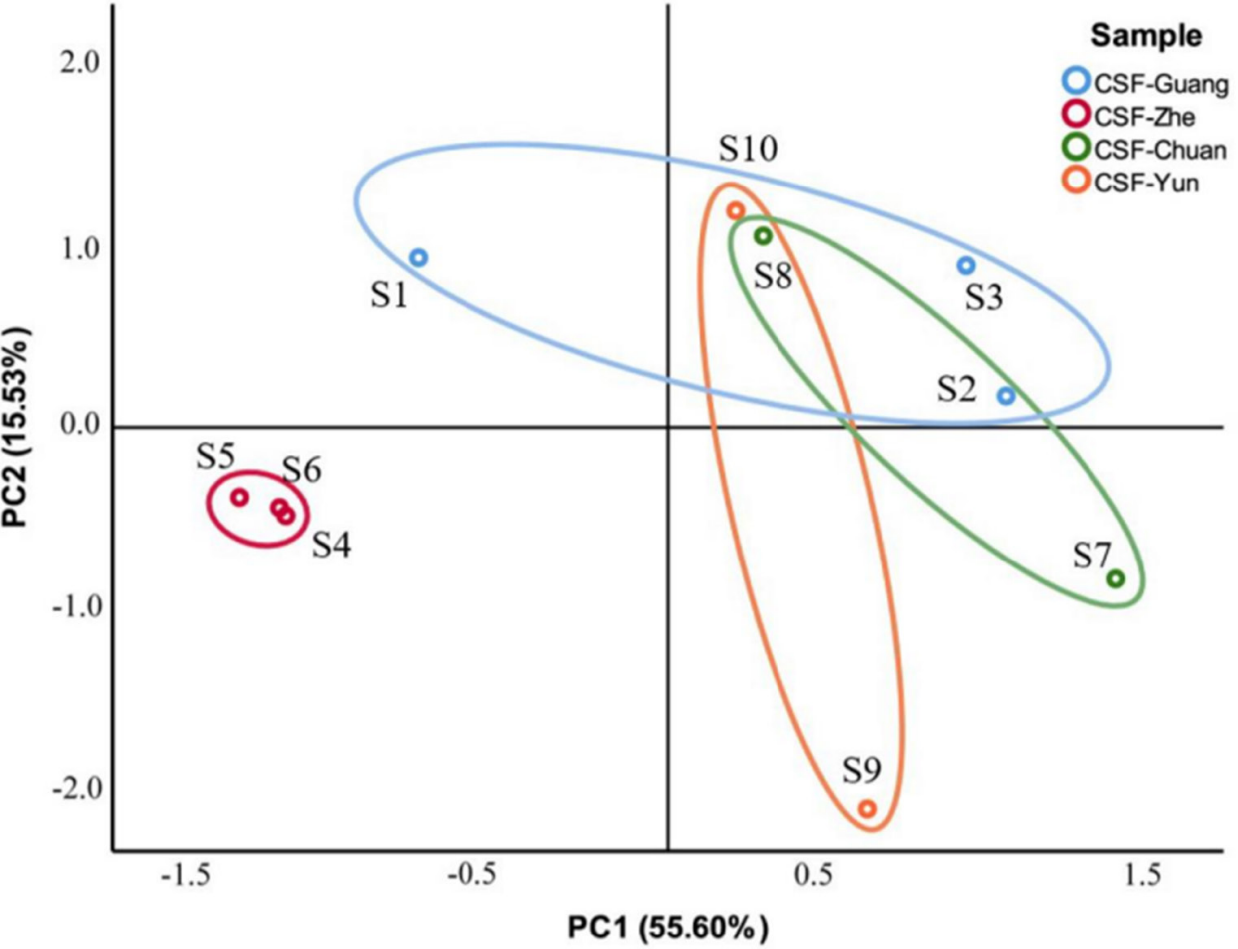
Multivariate analysis for major volatile components in CSF

To determine the difference of CSFs, principal component analysis (PCA) was performed on 35 major volatiles in the CSF samples. Consequently, five principal components (PCs) were extracted in total, with a cumulative contribution rate of 95.48% (Table 6). These five PCs represented 95.48% information of the 35 main volatile components. From the results, 55.60% of the variance was attributed to the first PC (PC1) and 15.53% was attributed to the PC2. PC1 was mainly related to D‐limonene, (‐)‐isolongifolol, (‐)‐spathulenol, (‐)‐terpinen‐4‐ol, ledol, t‐cadinol, α‐bisabolol, α‐terpineol, cubenol, methyl linoleate, methyl palmitate, (E)‐β‐ocimene, α‐terpinene, germacrene D, β‐pinene, β‐bisabolene, ɑ‐pinene, sabinene, thujene, α‐bergamotene, d‐cadinene, neoisolongifolene 8‐bromo‐, α‐caryophyllene, caryophyllene, β‐elemene, and β‐myrcene. The main contributors to PC2 were γ‐terpinene, nerol, linalool, citronellol, and citral. γ‐Elemene contributed to PC3. Neryl acetate and elixene contributed to PC4. p‐Cymene contributed to PC5. A PCA plot is shown in Figure 5. From the PCA plot, CSF‐Chuan and CSF‐Yun mainly distributed on the first and the fourth quadrant and CSF‐Guang (S2 and S3) distributed on the first quadrant. All samples of CSF‐Zhe were mainly concentrated on the third quadrant and CSF‐Guang (S1) distributed on the second quadrant, which indicates an obvious difference between CSF‐Zhe and CSF‐Guang (S1) with other origins, which is closely related to PC1. Overall, these results were in accordance with clustering analysis.

**TABLE 6:** Total variance explained of PCA of CSF from different origins

Component	Total	% of Variance	Cumulative %
1	19.46	55.60	55.60
2	5.44	15.53	71.13
3	3.92	11.19	82.32
4	2.80	7.99	90.31
5	1.81	5.17	95.48

## 4 CONCLUSIONS

In this study, a systemic qualitative and quantitative analysis of nonvolatile and volatile components was conducted. A particularly high content of stachydrine was detected in CSF, and it was the first time to performed stachydrine to quantitative analysis. A new referential index “5,7‐dimethoxycoumarin” was demonstrated to assess the quality and differences among CSF samples from various origins. CSF‐Zhe contained the most abundant 5,7‐dimethoxycoumarin and volatile oil in the four main origins. In addition, some important alkenes in CSF‐Zhe were significantly high, such as D‐limonene, γ‐terpinene, ɑ‐pinene, and β‐pinene, which indicated that there were certain chemical composition advantages in CSF‐Zhe. From the result, tetratetracontane was found to be detected in CSF‐Guang (S1), and terpinolene may be one of the characteristic compounds to distinguish CSF‐Zhe from other CSFs in this study. In summary, this work provided a comprehensive analysis method in CSFs, which conveyed a better understanding and sufficient reference for their further utilization.

## CONFLICTS OF INTEREST

The authors declare no conflicts of interest.

## AUTHOR CONTRIBUTION

**Guodong Zheng:** Methodology (equal); Software (equal); Supervision (equal); Validation (equal); Writing – review & editing (equal). **Jinrong Ning:** Conceptualization (equal); Investigation (equal); Visualization (equal); Writing – original draft (equal). **Kanghui Wang:** Conceptualization (equal); Investigation (equal); Visualization (equal); Writing – original draft (equal). **Wanling Yang:** Data curation (equal); Methodology (equal); Software (equal). **Mengshi Liu:** Data curation (equal); Methodology (equal); Software (equal). **Jingyuan Tian:** Data curation (equal); Methodology (equal); Software (equal). **Minyan Wei:** Methodology (equal); Software (equal); Supervision (equal); Validation (equal); Writing – review & editing (equal).

## ETHICAL APPROVAL

This study does not involve any human or animal testing.

## INFORMED CONSENT

Written informed consent was obtained from all study participants.
